# Aging Reduces ATP-Binding Cassette Transporter Expression in Brain Microvessels of Mice

**DOI:** 10.3390/ph18020191

**Published:** 2025-01-30

**Authors:** Yukiyo Wada, Masaki Inoko, Kanako Ishihara, Karin Fukumoto, Yuya Tsurudome, Michiko Horiguchi, Akio Fujimura, Kentaro Ushijima

**Affiliations:** 1 Division of Pharmaceutics, Faculty of Pharmaceutical Sciences, Sanyo-Onoda City University, Yamaguchi 756-0884, Japantsurudome-y19@rs.socu.ac.jp (Y.T.); horiguchi-michiko@rs.socu.ac.jp (M.H.);; 2 Department of Pharmaceutical Engineering, Sanyo-Onoda City University, Yamaguchi 756-0884, Japan; 3 Division of Clinical Pharmacology, Jichi Medical University, Tochigi 329-0498, Japan

**Keywords:** brain microvessel, transporter, aged, circadian rhythm

## Abstract

**Background:** ATP-binding cassette (ABC) transporters are expressed in the vascular walls of brain capillaries and remove toxic chemicals from the brain. The expression of ABC transporters in peripheral organs is transcriptionally regulated by clock genes and exhibits 24 h periodic fluctuations. In addition, clock gene outputs diminish with aging. In this study, we evaluated whether the expression of ABC transporters in the blood–brain barrier (BBB) of young mice had a 24 h cycle, and whether the expression of ABC transporters in the BBB decreased with age. **Methods:** Brain microvascular (BMV) fractions from the cerebral cortex of male C57BL/6J mice were prepared using dextran. BMV fractions from young mice (12 weeks old) were prepared every four hours to evaluate 24 h rhythmicity. BMV fractions from both young and aged mice (85 weeks old) were prepared when protein expression peaked (Zeitgeber Time 5). Protein and mRNA expression of ABC transporters in BMV fractions were measured. **Results:** In young mice, protein expression of P-glycoprotein, breast cancer resistance protein, and multidrug resistance protein 4 showed time-dependent variations with a peak in the light phase (Zeitgeber Time 5); mRNA expression showed no time-dependent variation. The protein expression of these transporters was lower in the BBB of aged mice than in that of young mice, although mRNA expression did not differ between young and aged mice. **Conclusions:** ABC transporter protein expression levels in BMV endothelial cells decreased with aging; however, mRNA levels did not change, which suggests changes in protein expression did not result from diminished clock gene output. Further studies are needed to elucidate the mechanisms by which ABC transporter expression in the BBB decreases with aging.

## 1. Introduction

The activity of several pharmacokinetic-related molecules is altered with aging. For example, the metabolism of CYP3A4 substrates is decreased in older people [1]; in addition, hepatic *Oatp1a1* and *Oct2* mRNA levels are reduced in aged mice [2]. The decreased function of these metabolic pathways could lead to the elevation of blood drug concentrations. For example, a clinical study showed that metoclopramide, a substrate of P-glycoprotein, accumulated more readily in brain tissue and was excreted into the bloodstream at lower levels in older people than in young subjects [3]. The blood–brain barrier (BBB) restricts the migration of drugs from peripheral blood to the brain. The BBB undergoes many pathological changes during the aging process including alterations to its structure, which, in turn, impede its highly selective dynamic barrier function [4]. Thus, P-glycoprotein expression and function in the BBB may be reduced in older people. Although transporter expression changes in peripheral organs during aging have been reported, little is known about transporter expression in the BBB.

ATP-binding cassette (ABC) transporters in the BBB utilize ATP to actively expel endogenous chemicals and drugs, primarily from the brain parenchyma into the vascular lumen [5]. Transporter members of the ABC superfamily include P-glycoprotein (encoded by *Abcb1a*), breast cancer resistance protein (BCRP; encoded by *Abcg2*), and multidrug resistance protein (encoded by *Abcc*) [6,7]. ABC transporters such as P-glycoprotein are highly expressed in the vascular walls of brain capillaries and play a major role in drug kinetics, efficacy, and toxicity in the brain [8]. In addition, recent studies have reported that ABC transporters play important roles in the brain transport of beta-amyloid peptide, which is a key factor in Alzheimer’s disease pathogenesis [9,10]. Therefore, down-regulation of ABC transporters in the BBB could elevate the risk of adverse drug reactions in the central nervous system and increase Alzheimer’s disease pathogenesis.

In mammals, daily rhythms in behavior and physiology are regulated by a circadian clock system. Circadian oscillators driven by clock gene products operate via self-sustained transcriptional/translational feedback loops [11]; this mechanism integrates positive and negative feedback from circadian clockwork circuitry to control 24 h variations in physiology. The expression and function of ABC transporters in mouse peripheral organs also exhibit 24 h periodic fluctuations [12,13,14,15]. Intestinal P-glycoprotein expression is transcriptionally regulated by the expression cycle of the clock genes E4 promoter binding protein 4 (encoded by *Nfil3*) and hepatic leukemia factor (HLF) [13]. The circadian clock system also coordinates the daily rhythmic expression of intestinal BCRP by the clock-controlled gene activating transcription factor 4, and MRP2 by clock genes such as albumin D-site-binding protein (DBP) and E4 promoter-binding protein 4 [12,14,15]. In addition, the efflux activity of MRP4 expressed in the rat cerebral choroid plexus exhibits 24 h rhythmicity [16]. Therefore, the transcriptional system of the circadian clock in peripheral tissues regulates daily rhythms in the expression and function of ABC transporters.

As the output signals of clock genes diminish with aging [17], the expression level of ABC transporters may be lower in older people because of reduced clock functionality in the BBB. However, information about the rhythmic pattern of ABC transporter expression in the BBB is limited [18]. In this study, we evaluated whether (1) the expression of ABC transporters in the BBB of young mice exhibited a 24 h cycle, and (2) the expression of ABC transporters in the BBB decreased with age.

## 2. Results

### 2.1. Rhythmic Expression of ABC Transporters in the BMV Fraction

**Figure 1:**
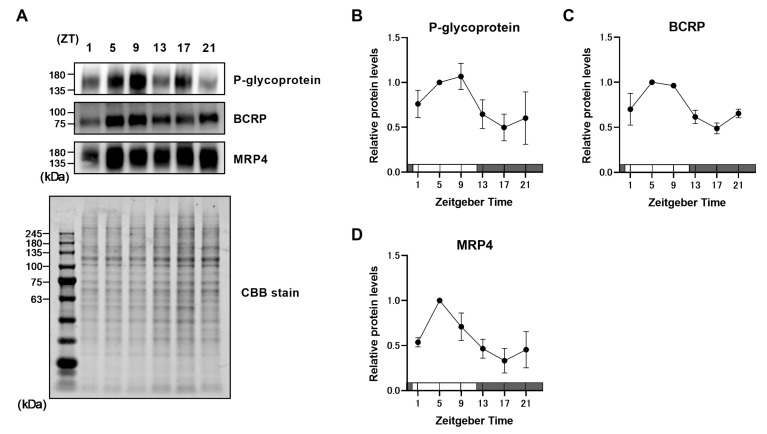
Daily rhythms of ABC transporter protein expression in the BMV fraction of 12-week-old male mice. (**A**) The upper panel shows Western blot images, and the lower panel shows a CBB stain image. (**B**–**D**) Daily rhythms in the protein expression levels of (**B**) P-glycoprotein, (**C**) BCRP, and (**D**) MRP4. The maximum expression level was set to 1.0; data are expressed as mean ± SEM from three independent measurements. Open bar, the light phase; closed bar, the dark phase.

**Figure 2:**
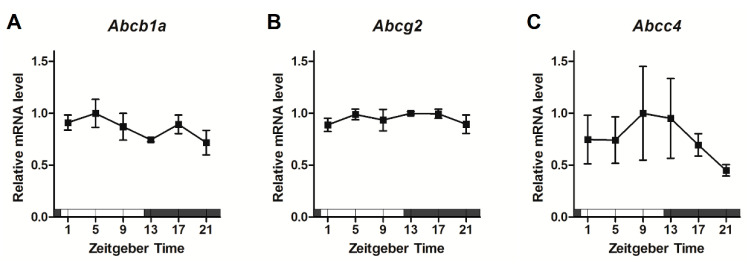
Daily rhythms of ABC transporter mRNA expression in the BMV fraction of 12-week-old male mice. (**A**–**C**) Daily rhythms in the mRNA expression levels of (**A**) *Abcb1a*, (**B**) *Abcg2*, and (**C**) *Abcc4*. The maximum expression level was set to 1.0; data are expressed as mean ± SEM, *n* = 3. Open bar, the light phase; closed bar, the dark phase.

To confirm the time-dependent expression of ABC transporters, we obtained BMV fractions from 12-week-old mice at the following six time points: Zeitgeber Time (ZT) 1, 5, 9, 13, 17, and 21 (ZT0: lights on; ZT12: lights off). Protein expression of P-glycoprotein, BCRP, and MRP4 showed time-dependent variations with a peak around ZT5–9 and a trough at ZT17 (Figure 1A–D). MRP2 protein expression in the BMV fraction was too low to quantify under the present conditions. By contrast, mRNA levels of *Abcb1a*, *Abcg2*, and *Abcc4* did not show time-dependent variations in their expression (Figure 2A–C). Statistical analysis using the cosinor method revealed significant daily rhythms in the protein expression of P-glycoprotein, BCRP, MRP4, and *Dbp* but not in their mRNA expression (Table 1).

### 2.2. Rhythmic Expression of Clock Genes in the BMV Fraction

**Figure 3:**
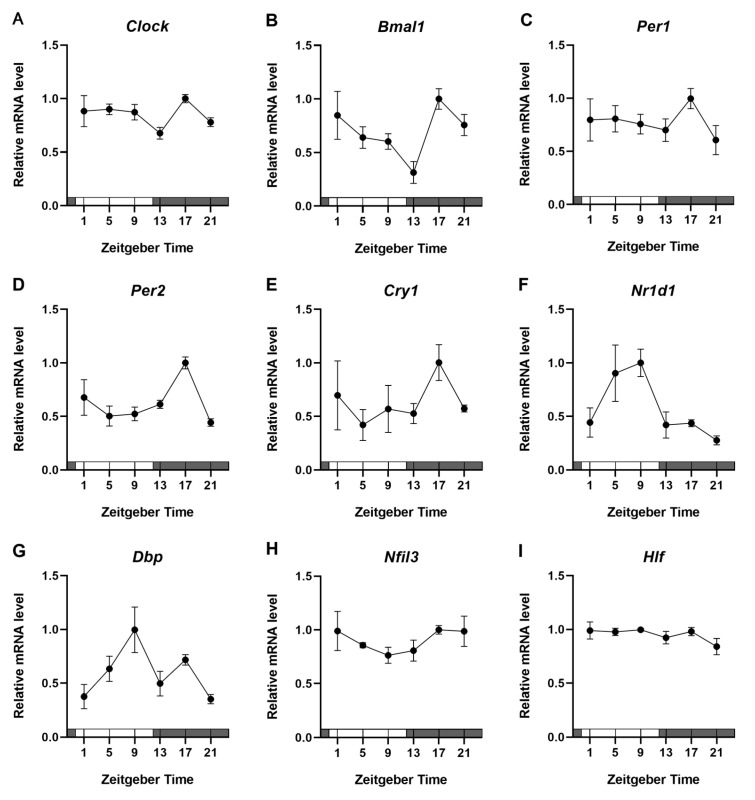
Daily expression profile of clock genes in the BMV fraction of 12-week-old male mice. Daily rhythms of the mRNA expression of the clock genes (**A**) *Clock*, (**B**) *Bmal1*, (**C**) *Per1*, (**D**) *Per2*, (**E**) *Cry1*, (**F**) *Nr1d1*, (**G**) *Dbp*, (**H**) *Nfil3*, and (**I**) *Hlf*. The maximum expression level was set to 1.0; data are expressed as the mean ± SEM, *n* = 3. Open bar, the light phase; closed bar, the dark phase.

To verify the rhythmic expression of clock genes in the BBB, we prepared BMV fractions from 12-week-old mice at six time points. The mRNA expression levels of the clock genes *Clock*, *Bmal1*, *Per1*, *Per2*, *Cry1*, *Nr1d1*, *Dbp*, *Nfil3*, and *Hlf* were measured. Overall, the amplitudes of rhythmic expression were weak (Figure 3A–I). *Clock*, *Bmal1*, *Per1*, *Per2*, and *Cry1* mRNA expression peaked during the dark phase (active period), while *Nr1d1* and *Dbp* mRNA expression peaked during the light phase (resting period). *Nfil3* and *Hlf* did not exhibit rhythmic expression patterns. Statistical analysis using the cosinor method revealed significant daily rhythms in *Bmal1*, *Per2*, *Nr1d1*, and *Dbp* mRNA expression (Table 2). The expression of other clock genes, such as *Clock*, *Per1*, *Cry1*, and *Nfil3*, tended to show periodicity but rhythmicity was not detected in *Hlf* mRNA.

### 2.3. Aging Alters ABC Transporter Expression in BMV

**Figure 4:**
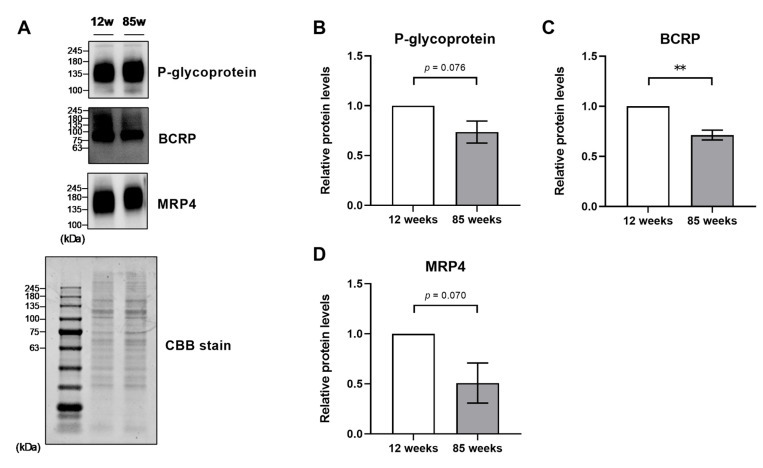
Influence of aging on the protein expression of ABC transporters in the BMV fraction. The BMV fraction was obtained at ZT5. (**A**) The upper panel shows Western blot images, and the lower panel shows a CBB stain image. (**B**–**D**) Daily rhythms in the protein expression levels of (**B**) P-glycoprotein, (**C**) BCRP, and (**D**) MRP4. The expression level in 12-week-old mice was set to 1.0; data are expressed as mean ± SEM from three independent measurements, ** *p <* 0.01.

**Figure 5:**
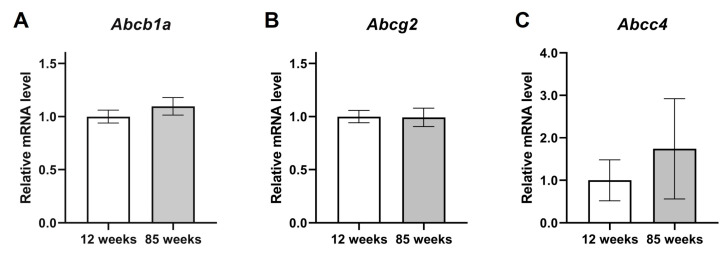
Influence of aging on the mRNA expression of ABC transporters in the BMV fraction. (**A**–**C**) The mRNA expression levels of (**A**) *Abcb1a*, (**B**) *Abcg2*, and (**C**) *Abcc4*. The BMV fraction was obtained at ZT5. The expression level in 12-week-old mice was set to 1.0; data are expressed as mean ± SEM, *n* = 3.

To investigate the influence of aging on ABC transporter expression, we compared the expression between 12-week-old (young) mice and 85-week-old (aged) mice. In this experiment, we evaluated the BMV fraction only at ZT5 because the expression level of P-glycoprotein and BCRP peaked around ZT5–9, and MRP4 peaked at ZT5 in young mice as described above. The protein level of BCRP in aged mice was significantly lower than that in young mice (*p* < 0.01), and the levels of P-glycoprotein and MRP4 in aged mice were approximately 30% lower than those in young mice (Figure 4A–D). By contrast, the mRNA levels of *Abcb1a* and *Abcg2* were not significantly different between young and aged mice (Figure 5A,B). The mRNA levels of *Abcc4* in aged mice tended to be higher than in young mice, but the difference was not statistically significant (Figure 5C).

## 3. Discussion

In this study, we first measured the protein and mRNA expression of ABC transporters in BMV fractions from young mice. No significant rhythm was detected in the mRNA expression of any ABC transporters (Figure 2). In a previous report, no rhythmic pattern was observed in the expression of ABC transporter genes in the BMV fraction [18], similar to the present findings. In contrast, protein expression of the ABC transporters showed a diurnal rhythm with a peak around ZT5–9 and a trough at ZT17 (Figure 1). These rhythmic expression patterns were similar to those of ABC transporters in mouse small intestine reported previously [12,14]. In addition, the oscillatory regulation of gene transcription by clock genes was intact in mouse BMV fractions (Figure 3). Based on these observations, we speculate that the daily rhythmic protein expression of ABC transporters in mouse BMV endothelial cells is not dependent on transcriptional regulation by clock genes but rather on daily variation in post-translational modification. Specifically, we hypothesize that the daily rhythm of ABC transporter protein expression may be regulated via translation rate by Mg^2+^. Cell-autonomous clock regulation of the Mg^2+^ transporter transient receptor potential melastatin-subfamily member 7 causes cyclic changes in free Mg^2+^ concentrations in brain microvascular endothelial cells (BMVECs) [18]; these fluctuations contribute to the periodic regulation of protein translation rate independent of clock genes [19]. Thus, daily changes in Mg^2+^ concentration in BMVECs might be one mechanism responsible for the daily rhythm of ABC transporter protein expression, rather than transcriptional regulation by the clock system.

In a previous study, protein expression of P-glycoprotein in the BBB was lower in aged (36-month-old) rats than in young (3-month-old) rats [20]. The 85-week-old mice used in this study are equivalent to 79-year-old humans [21], while 36-month-old rats are equivalent to 80-year-old humans [22]. In this study, we compared ABC transporter expression between young and aged mice at ZT5 when higher ABC transporter protein expression was detected in young mice. Protein expression levels of P-glycoprotein, BCRP, and MRP4 in the BMV fraction of aged mice were lower than those of young mice (Figure 4). However, there was no significant difference in the mRNA expression of each ABC transporter between the young and aged mice (Figure 5). Therefore, although output signals by clock genes diminish with aging [17], the influence of clock genes on the protein expression of ABC transporters in BMVECs appears to be small.

Expression values of *Abcc4* (Figure 2C and Figure 5C) and *Cry1* (Figure 3C) mRNA in the BMV fractions showed large inter-assay variability. Generally, *Abcc4* mRNA is highly expressed in the kidney and leukocytes, while its expression in the vascular endothelium is less prominent [23,24]. Regarding *Cry1*, the abundance of mRNA quantified by the qRT-PCR method in this study was much lower than the other clock genes assayed. Therefore, the low expression of *Abcc4* and *Cry1* may have caused the large inter-assay variations we observed.

Many molecular biology studies report post-transcriptional regulation of P-glycoprotein expression. For example, the expression of protein phosphatase 2 regulatory subunit B″gamma in mouse liver decreases with aging [25]. Protein phosphatase 2 regulatory subunit B″gamma suppresses P-glycoprotein dephosphorylation; therefore, this age-associated decrease results in increased P-glycoprotein expression [26]. In addition, mitogen-activated protein kinase kinase inhibition increases the downstream activity of ubiquitin-conjugating enzyme E2 R1, which promotes ubiquitination and degradation of P-glycoprotein [27]. In a previous study, the expression of mitogen-activated protein kinase kinase in the liver was higher in aged (22-month-old) mice than in young (3-month-old) mice [28]. Based on these observations, ubiquitination of P-glycoprotein in older people may be decreased, which would lead to an elevation in protein expression. However, P-glycoprotein expression was decreased in the BBB of aged mice in this study, indicating that the involvement of these cascades in BMVECs is small.

As mentioned above, the regulation of protein translation by Mg^2+^ may be involved in the daily rhythm of ABC transporter expression. In elderly individuals, abnormalities in electrolyte concentrations, including Mg^2+^, have been frequently detected [29]. In addition, a previous animal study reported that Mg^2+^ concentration in the cortex is reduced in aged mice [30]. These data lead us to speculate that Mg^2+^ concentration in the cerebral microvasculature in aged mice is lower than in young mice and that this causes a decrease in ABC transporter protein expression.

## 4. Materials and Methods

### 4.1. Animal Experiments

Male C57BL/6J mice (10 and 68 weeks of age) were purchased from Japan SLC Inc. (Shizuoka, Japan). Mice were housed in a specific-pathogen-free room at 24 ± 1 °C and humidity levels of 60% ± 10%; food and water were freely available. Animals were maintained under a 12 h light–dark cycle. The light-on time was ZT0. To investigate the rhythmic expression of ABC transporters and clock genes, 36 mice aged 12 weeks old were used. After acclimatization over 2 weeks, brains were collected every 4 h (ZT1, 5, 9, 13, 17, and 21). Three mice were used for protein and RNA measurement at each sampling point. To investigate alterations in ABC transporter expression in aged mice, six mice aged 12 weeks old and six mice aged 85 weeks old were used. After acclimatization over 2 weeks, brains were collected at ZT5. Three mice were used for protein and RNA measurement; specimens from three different mice were pooled and treated as one sample to obtain enough protein for Western blotting in both experiments. All experiments were conducted in accordance with a protocol approved by the Internal Committee for Animal Experiments at Sanyo-Onoda City University (ethical approval protocol IC: #A-2024-52-A, 10 May 2024).

### 4.2. Preparation of Brain Microvascular Fractions

Preparation of mouse BMV fractions was performed using centrifugation with dextran buffer, as reported previously, which reduces denaturation and damage to protein and RNA [31]. Brains were isolated from mice under anesthesia and washed with ice-cold Hank’s balanced salt solution. The arachnoid membranes were peeled from the excised brains, and the cerebral cortex was isolated. The isolated cerebral cortex was homogenized twice using a glass homogenizer with 1 mL of Hank’s balanced salt solution and transferred to a centrifuge tube. To obtain enough protein for Western blotting, specimens from three different mice were pooled and treated as one sample. After this, 4 mL of 26.5% dextran was added to the tube containing BMVECs at 4 °C and inverted. The homogenized suspension was centrifuged at 4300× *g* for 30 min at 4 °C. The pellet containing BMVECs was suspended in 3 mL of 17.7% dextran and centrifuged at 4300× *g* for 30 min at 4 °C. The pellet was resuspended with 1 mL of phosphate-buffered saline and centrifuged at 5400× *g* for 5 min at 4 °C. The obtained pellet was frozen and stored at −80 °C as the BMV fraction.

### 4.3. RNA Extraction and cDNA Synthesis

Total RNA was extracted from the BMV fraction using a PureLink RNA Mini Kit (Thermo Fisher Scientific, Waltham, MA, USA), following the manufacturer’s instructions. RNA was eluted with 30 μL RNase-free water and quantified using a NanoDrop One system (Thermo Fisher Scientific). Complementary DNA was synthesized using a PrimeScript RT Reagent Kit (Takara Bio, Shiga, Japan), following the manufacturer’s instructions, and stored at −20 °C.

### 4.4. Quantitative Real-Time PCR (qRT-PCR)

cDNA equivalent to 100 ng of RNA was amplified using a StepOne Plus Real-Time PCR System (Thermo Fisher Scientific) with TB Green Premix Ex Taq II (Takara Bio), following the manufacturer’s instructions. The sequences of forward and reverse primers are displayed in Table 3. The relative level of each product was normalized to 18S rRNA (18rs). Data were analyzed by the comparative Ct method.

### 4.5. Protein Extraction

Protein was extracted by homogenization with 200 μL of RIPA buffer containing 50 mM Tris-HCl buffer, 150 mM NaCl, 1% nonidet P40 substitute, 0.5% sodium deoxycholate, and 0.1% sodium dodecyl sulfate. The concentration of protein lysate was quantified by a DC protein assay (Bio-Rad Laboratories, Hercules, CA, USA) and adjusted to 0.79 μg/μL. The protein lysate was combined with 2% sodium dodecyl sulfate and 1% 2-mercaptoethanol and denatured at 95 °C for 5 min.

### 4.6. Western Blotting

Denatured protein samples containing 3.95 μg protein were separated by sodium dodecyl sulfate-polyacrylamide gel electrophoresis and transferred to a polyvinylidene difluoride membrane. Separated proteins were stained with Coomassie Brilliant Blue as a control to verify equal membrane fraction amounts. The membrane was blocked with 1% nonfat dry milk (Cell Signaling Technology, Danvers, MA, USA) for 1 h at room temperature and incubated with primary antibodies diluted with Can Get Signal Solution 1 (TOYOBO, Osaka, Japan) overnight at 4 °C. Secondary antibodies diluted with Can Get Signal Solution 2 (TOYOBO) were added to the membrane and incubated for 1 h at room temperature. Protein was detected with Clarity Western ECL Substrate (Bio-Rad Laboratories) and ImmunoStar LD (FUJIFILM Wako Pure Chemical Corporation, Osaka, Japan) using a ChemiDoc Touch Imaging System (Bio-Rad Laboratories). The antibodies used for Western blotting are displayed in Table 4.

### 4.7. Statistical Analysis

Rhythmicity was analyzed by the cosinor method [32,33]. Differences between two groups were evaluated using a two-tailed unpaired *t*-test (GraphPad Prism version 8.4.3, San Diego, CA, USA). The peak time was determined as the time when protein and mRNA expression was highest. All data are expressed as mean ± SEM, and a *p*-value less than 0.05 was considered statistically significant. The sample size was not predetermined and was based on previous studies. In Western blotting analysis, intensities of the target protein band and the bands of Coomassie Brilliant Blue staining were calculated using Image J software (version 13.0.6, https://imagej.net/ij/).

## 5. Conclusions

This study demonstrates that protein expression levels of P-glycoprotein, BCRP, and MRP4 are lower in the BBB of aged mice than in that of young mice. Many studies have investigated the physiological regulation of ABC transporter expression in aged subjects [34,35]; however, the conclusions are inconclusive. Although the present study could not clarify exact molecular mechanisms, translation rate regulation by Mg^2+^ might underlie the discrepancy between mRNA and protein expression in both daily rhythmic patterns and aging. Further studies are needed to elucidate the mechanisms by which ABC transporter expression in the BBB decreases with aging, which could help to develop a novel medicine for age-related neurological disorders such as Alzheimer’s disease. In addition, a quantitative evaluation of changes in drug transport into the brain resulting from aging would enable appropriate drug treatment regimens for older people to be formulated.

## Figures and Tables

**Table 1:** Statistical analysis of ABC transporter expression by the cosinor method. Bold style, statistically significant.

**Protein**	**Amplitude**	**Mesor**	**Acrophase (h)**	**Period (h)**	***p* Value**	**F Value**
P-glycoprotein	0.304	0.772	9.30	21.6	**0.0194**	5.15
BCRP	0.282	0.746	9.42	20.5	**0.0003**	17.51
MRP4	0.312	0.588	9.86	21.3	**0.0036**	8.578
**Gene**	**Amplitude**	**Mesor**	**Acrophase (h)**	**Period (h)**	***p* Value**	**F Value**
*Abcb1a*	0.0881	0.8561	11.87	28	0.2973	1.316
*Abcg2*	0.0534	0.9419	4.01	28	0.5420	0.831
*Abcc4*	0.2576	0.724	6.5	28	0.3124	1.259

**Table 2:** Statistical analysis of clock gene expression by the cosinor method. Bold style, statistically significant.

Gene	Amplitude	Mesor	Acrophase (h)	Period (h)	*p* Value	F Value
*Clock*	0.117	0.862	9.25	15.0	0.0506	3.64
*Bmal1*	0.251	0.669	11.09	18.9	**0.0341**	4.24
*Per1*	0.133	0.781	4.54	15.0	0.1880	1.87
*Per2*	0.221	0.618	10.46	17.5	**0.0129**	5.88
*Cry1*	0.219	0.619	3.56	19.2	0.1932	1.83
*Nr1d1*	0.404	0.601	18.00	20.0	**0.0010**	12.14
*Dbp*	0.323	0.579	13.18	18.2	**0.0027**	9.73
*Nfil3*	0.131	0.900	11.42	23.7	0.1026	2.64
*Hlf*	0.045	0.953	17.91	24.7	0.5894	0.951

**Table 3:** Primer information used for qRT-PCR.

Gene Name	Accession ID	Sequences
*Clock*	NM_007715	Forward: AACCGTAGCAGGTTTATGGGAATG Reverse: TTGGTGTCCACACAATAGGCAAG
*Bmal1*	NM_007489	Forward: ACGACATAGGACACCTCGCAGA Reverse: CGGGTTCATGAAACTGAACCATC
*Per1*	NM_011065	Forward: GTCTGGTTCAGGATCCCACGA Reverse: TGCTGCCAAAGTACTTGCTTGTATG
*Per2*	NM_011066	Forward: ATCAGCCATGTTGCCGTGTC Reverse: CGTGCTCAGTGGCTGCTTTC
*Cry1*	NM_007771	Forward: GGATCCACCATTTAGCCAGACAC Reverse: CATTTATGCTCCAATCTGCATCAAG
*Nr1d1*	NM_145434	Forward: TGCTTAAGGCTGGCACCTTTG Reverse: GTAGGTTGTGCGGCTCAGGAA
*Dbp*	NM_016974	Forward: AAGCATTCCAGGCCATGAGAC Reverse: TTCTTGTACCTCCGGCTCCAG
*Nfil3*	NM_017373	Forward: CAAATCGGAACACTGGCATCAC Reverse: AGCCACCGTCTTTGACTTCCAC
*Hlf*	NM_172563	Forward: TGACCTGCCGTGAGTTGTAAGTG Reverse: GGAATGCTAAGGGCCAAACTGA
*Abcb1a*	NM_011076.3	Forward: GTCCCAACTGGGATATTGTACA Reverse: AGGTGCCCATGTCTGAGTAA
*Abcc4*	NM_001033336.3	Forward: CCTGGAATCCACAACACGGA Reverse: TTTGTAAGCCCGGATGGTCC
*Abcg2*	NM_011920.3	Forward: AGCTCCGATGGATTGCCAG Reverse: GAGGGTTCCCGAGCAAGTTT
*18rs*	NM_011296	Forward: TTCTGGCCAACGGTCTAGACAAC Reverse: CCAGTGGTCTTGGTGTGCTGA

**Table 4:** Antibody information used for Western blotting.

**Primary Antibody (Host)**	**Dilution**	**Supplier**	**Catalog No.**
Anti-P-glycoprotein (Rabbit)	1:1000	Proteintech	22336
Anti-BCRP (Rat)	1:1000	Santa Cruz Biotechnology	sc-58224
Anti-MRP4 (Rat)	1:1000	Abcam	ab15602
**Secondary Antibody (Host)**	**Dilution**	**Supplier**	**Catalog No.**
HRP-linked horse anti-mouse IgG	1:10,000	Cell Signaling Technology	7076
HRP-conjugated Goat anti-rat IgG	1:10,000	Santa Cruz Biotechnology	sc-2032

## Data Availability

Data from this study are available from the corresponding author upon reasonable request.
